# Does visual speech provide release from perceptual masking in children?

**DOI:** 10.1121/10.0001867

**Published:** 2020-09-04

**Authors:** Destinee M. Halverson, Kaylah Lalonde

**Affiliations:** Center for Hearing Research, Boys Town National Research Hospital, Omaha, Nebraska 68104, USA halverd@wwu.edu, kaylah.lalonde@boystown.org

## Abstract

Adults benefit more from visual speech in speech maskers than in noise maskers because visual speech helps perceptually isolate target talkers from competing talkers. To investigate whether children use visual speech to perceptually isolate target talkers, this study compared children's speech recognition thresholds in auditory and audiovisual condition across two maskers: two-talker speech and noise. Children demonstrated similar audiovisual benefit in both maskers. Individual differences in speechreading accuracy predicted audiovisual benefit in each masker to a similar degree. Results suggest that although visual speech improves children's masked speech recognition thresholds, children may use visual speech in different ways than adults.

## 1. Introduction

Listening in noise is challenging for adults and children, but children are especially susceptible to masking [see Leibold and Buss (2019) for review]. Developmental differences in susceptibility to masking are particularly pronounced when background noise consists of a small number of competing talkers. In such conditions, children must rely on incompletely developed central auditory and cognitive processes and language skills to perceptually group acoustic cues from each talker into separate auditory objects and selectively attend to the talker of interest (Brungart, 2001; Leibold and Buss, 2019). Certain stimulus differences between target and masker speech promote acoustic grouping in both children and adults, resulting in improved speech-in-speech recognition. These include differences in language, the typical vocal characteristics of male and female talkers (i.e., fundamental and resonant frequencies), and spatial location (Leibold and Buss, 2019; Litovsky, 2005).

Visual speech is a stimulus characteristic known to promote grouping in adults (Helfer and Freyman, 2005). Adults benefit more from visual speech cues in the presence of speech maskers than in spectrally matched noise maskers (Avivi-Reich *et al.*, 2018; van Engen *et al.*, 2017; Helfer and Freyman, 2005). Whereas visual speech provides supplemental phonetic information in both maskers (i.e., visual cues to place of articulation when acoustic place cues are masked), visual speech also helps adults to perceptually separate target speech from the mixture of voices in the speech masker (Helfer and Freyman, 2005). Visible movements of the articulators correlate over time with spectro-temporal fluctuations in the acoustic speech signal (Chandrasekaran *et al.*, 2009). The visual signal reduces target/masker uncertainty in speech maskers by providing cues as to which acoustic intensity fluctuations are part of the target signal and which are part of the masker (Zion Golumbic *et al.*, 2013).

Children also benefit from visual speech cues in the context of masked speech recognition [e.g., Lalonde and McCreery (2020) for review (Ross *et al.*, 2011)]. As young as 4 years of age, children have sufficient visual phonetic knowledge to use supplemental phonetic information from visual speech (Lalonde and Holt, 2015). However, it is unclear whether children can specifically use visual speech to help perceptually isolate a target talker. Lalonde and McCreery (2020) completed the first study to address this question. Children need help perceptually isolating target talkers in speech maskers, but not in noise maskers (Leibold *et al.*, 2016). Therefore, Lalonde and McCreery (2020) examined school-age children and adults' auditory and audiovisual (AV) speech perception across a speech-spectrum noise masker (SSN) and a two-talker speech masker (TTS). Participants were assessed using two outcome measures: syllable detection thresholds and sentence recognition accuracy. AV benefit to syllable detection was five times greater in the TTS than in the SSN in children and adults alike. In contrast with previous adult studies [e.g., Helfer and Freyman (2005)], AV benefit to speech recognition accuracy was similar in the TTS and in the SSN for both children and adults. One potential explanation for this finding is that performance was not equated across maskers in the auditory-only baseline condition of the speech recognition task. Given that AV speech recognition benefit varies across levels of auditory performance (Ross *et al.*, 2011), the authors posited that any increased speech recognition benefit in the TTS may have been concealed by differences in auditory baseline performance. To determine whether children use visual speech to perceptually isolate target talkers, the authors suggested it might be necessary to avoid differences in auditory baseline by measuring children's sentence recognition *thresholds* in each masker and modality.

The purpose of the present study was to determine whether children use visual speech to help perceptually isolate a target talker. To that end, we compared auditory and AV speech recognition thresholds across a TTS and an SSN. Additionally, we examined the relationship between individual differences in children's ability to extract information from visual-only (VO) speech and their AV benefit in each masker. We expected children in this age range to benefit in both maskers, because children can use supplemental phonetic information from visual speech (Lalonde and Holt, 2015). Children need help perceptually isolating target talkers in a TTS but not in an SSN (Leibold *et al.*, 2016). Therefore, if children use visual speech to perceptually isolate target talkers, we expected to observe two differences in results across maskers. First, like adults [e.g., Helfer and Freyman (2005)], we expected children to demonstrate added AV benefit in the TTS relative to the SSN. Second, we reasoned that whereas individual differences in children's AV benefit in an SSN relate to individual differences in their ability to extract phonetic information from visual speech, individual differences in children's AV benefit in a TTS are influenced by individual differences in both the ability to extract phonetic information from visual speech and the ability to use visual speech to perceptually isolate a target talker. Therefore, we expected that individual differences in speechreading accuracy—a proxy for the ability to extract phonetic information from visual speech—would relate more closely to AV benefit in the SSN than in the TTS.

## 2. Method

### 2.1 Participants

Fifteen children between 7 and 9 years of age (mean = 8.4, SD = 0.97, 10 female) and ten adults between 19 and 25 years of age (mean = 26.5, SD = 4.87, 7 female) participated in this experiment. One additional adult was excluded for failing to comply with experimenter instructions. Participants were native English speakers who passed a 20 dB hearing level pure-tone hearing screening bilaterally at octave intervals from 0.25 to 8 kHz (ANSI, 2004). Each participant also passed a vision screening for normal or corrected-to-normal visual acuity (at least 20/30 vision bilaterally) using a Snellen eye chart. Adult participants and child participants' parents reported no developmental concerns or color blindness.

### 2.2 Stimuli

Target stimuli were modeled after the Coordinate Response Measure (CRM) stimulus corpus (Bolia *et al.*, 2000). Each recording consists of the phrase “Now you will go to [color] [number]” spoken by a female talker. There were four colors (red, white, green, blue) and eight numbers (1–9, excluding 7). All videos began with a neutral face approximately 500 ms before the onset of the acoustic speech and ended with a neutral face approximately 300 ms after the talker's final mouth movement. Audio clips were modified in Adobe Audition to equate root mean square of each carrier phrase (i.e., “Now you will go to”). By modifying the amplitude of the entire signal based on only the carrier phrase, we preserved natural variability in amplitude across utterances with different phonetic content (range = 2.4 dB). Stimuli are available online (Lalonde and Halverson, 2020).

The maskers included a TTS consisting of two female talkers reading *Jack and the Beanstalk* (Walker, 1999) and an SSN with the same long-term average spectrum as the TTS. Both maskers were used in previous research by Calandruccio and colleagues (2016) and Lalonde and McCreery (2020).

### 2.3 Procedures

This research was approved by the Institutional Review Board at Boys Town National Research Hospital. Participants were compensated for their time at a rate of $15/h. Participants' speech recognition thresholds were measured in two modalities [auditory-only (AO) and AV] and two maskers (TTS, SSN) using a repeated measures design. Modality and masker order were counterbalanced across participants, except that each participant completed all testing in one masker before beginning testing in the second masker. In the AO condition, the visual stimulus was a still image of the talker's full neutral face throughout each stimulus interval. In the AV condition, the visual stimulus consisted of a synchronous, congruent video of the talker's full face.

Participants were seated inside a double-walled sound booth facing a 27-in. touch screen monitor. Auditory stimuli were presented via two speakers located at +/− 45° azimuth relative to the listener. The masker and target signal were presented simultaneously to both speakers, offering no perceived spatial separation. After each stimulus, a 32-color/number response grid appeared on the screen. Participants were instructed to touch the perceived color/number. No feedback was given. Custom software on a Mac Pro computer-controlled stimulus presentation and saved responses. The experimenter sat in the booth with children during testing and offered breaks between test blocks.

Testing for each condition included two phases: familiarization in quiet and test in noise. All participants responded correctly to five consecutive familiarization trials in quiet at 65 dB sound pressure level (SPL) before continuing to the test phase. In the test phase, an adaptive two-down/one-up procedure was used to estimate the signal-to-noise ratio (SNR) at which participants could provide the correct color/number with 70.7% accuracy (Levitt, 1971). Masker level was held constant at 65 dB SPL and signal level varied from trial to trial. Each test run began at +5 dB SNR with a 4 dB step size. Step size decreased to 2 dB after the second reversal in direction of SNR change and remained at 2 dB for the remaining ten reversals. To prohibit test runs from continuing indefinitely, testing ended before the tenth reversal if a participant responded correctly on ten trials in a row at the minimum SNR (−40 dB). This was considered ceiling performance, as we assumed correct responses at such poor SNRs would be based purely on speechreading. Threshold SNR was defined as the mean SNR at the final four reversals. Participants completed two consecutive test runs in each condition.

Participants also completed a visual-only (VO) condition, in which the videos from the AV condition were presented with no auditory signal. VO testing was completed after testing in either the first or second masker, with order counterbalanced across participants, so that participants had experience with task before completing VO testing. The VO condition began with a five-trial familiarization phase but proceeded to the test phase regardless of the accuracy of familiarization responses. The test phase consisted of two 30-trial blocks.

## 3. Results

### 3.1 Statistical approach

Statistical analyses were performed using rstudio (Version 1.1.456). Data were analyzed by fitting linear mixed models using the *lmer* and *anova* functions in the *lmerTest* package in r (Bates *et al.*, 2015; Kuznetsova *et al.*, 2017). The *anova* function provides F-statistics for models generated using the *lmer* function. Participant sex was initially included as a variable in analyses, but no significant effect of sex and no interactions of sex with other variables emerged. Therefore, this variable was removed. Six of ten adults reached ceiling in both AV conditions, so adults' AO and AV thresholds were only used for qualitative comparison.

### 3.2 Speech recognition thresholds

**Fig. 1.**
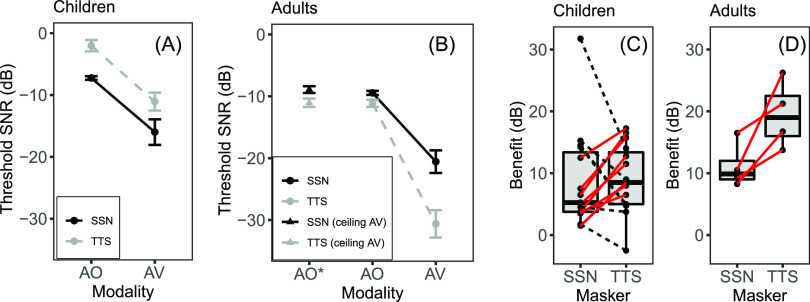
(Color online) (A) Children's mean speech recognition thresholds in an SSN (black) and a TTS (grey). Error bars show standard error of the mean. (B) Adult's mean speech recognition thresholds in SSN (black) and TTS (grey). Error bars show standard error of the mean. AO* indicates results for the 6 adults who reached ceiling in AV conditions; AO and AV indicate the 4 adults who did not reach ceiling in any condition. (C) Boxplot showing the distribution of children's AV benefit scores (AV – AO) in each masker, overlaid with individual data. Solid lines represent results for children who demonstrated greater benefit in TTS; dotted lines represent children who demonstrated greater benefit in SSN. (D) Boxplot showing the distribution of adults' AV benefit scores (AV – AO) in each masker, overlaid with individual data.

Figure 1(A) shows children's speech recognition thresholds in the SSN (black) and TTS (grey) across the two modalities. A mixed linear model was fit to analyze the fixed effects of masker type (TTS, SSN) and modality (AO, AV)—as well as the interaction among these variables—on speech recognition thresholds. A random intercept for each participant was included to account for correlations among repeated measures.

The effects of modality F_1,42_ = 49.84, p < 0.001, and masker F_1,42_ = 16.26, p < 0.001 were significant. On average, children's thresholds were 5.1 dB lower in the SSN than in the TTS and 9.1 dB lower in the AV condition than in the AO condition. There was no significant interaction, F_1,42_ = 0.01, p = 0.906, suggesting that benefit was the same regardless of masker type. Figure 1(C) shows individual AV benefit scores in each masker. Eight of the 15 children benefited more from visual speech in the TTS.^1^

For comparison, Figs. 1(B) and 1(D) show the same data for the ten adults. In Fig. 1(B), AO* shows AO threshold for the six adults who reached ceiling in the AV conditions. AO and AV include the four adults who did not reach ceiling. AO thresholds were similar across the two groups of adults. Among the four adults who did not reach ceiling in the AV conditions, mean AV benefit was 11.1 dB in the SSN and 19.5 dB in the TTS. Note that omitting adults who reached ceiling performance resulted in under-estimation of AV benefit in both maskers. All four adults benefited more from visual speech in the TTS than the SSN.

### 3.3 Visual-only accuracy

**Fig. 2.**
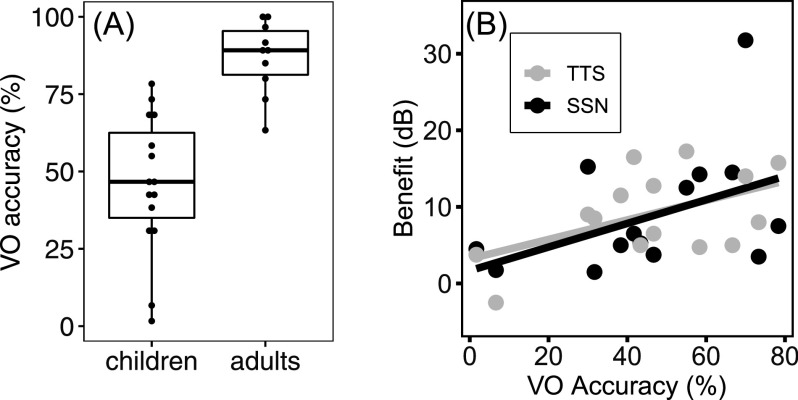
(A) Boxplot showing the distribution of VO accuracy scores in each age group. (B) Children's AV benefit in the SSN (black) and the TTS (grey) plotted as a function of their VO accuracy scores.

The boxplots in Fig. 2(A) show VO accuracy scores for all participants. Children identified the VO color-number coordinates with an average of 45.9% accuracy (S.D. = 22.5%). There was considerable variability, ranging from 1.7% to 78.3% correct. Adults identified the same stimuli with an average of 86.8% accuracy (S.D. = 11.8%). Whereas the six adults who reached ceiling in the AV condition had VO scores of 88% to 100% correct, the four adults who did not reach ceiling had VO scores of 63% to 85% correct. This suggests that the adults who reached ceiling in AV conditions could do so by relying exclusively on visual speech.

We examined whether individual differences in children's speechreading ability would account for individual variability in children's AV benefit (AV – AO) using a mixed linear model with effects of masker (TTS and SSN) and VO accuracy and a random intercept for participant. Results are shown in Fig. 2(B). There was a significant effect of visual-only accuracy, B = 0.14 dB, t = 2.556, p = 0.024, which did not interact with masker type. The model suggests a 1.4 dB increase in children's AV benefit for every 10% increase in their speechreading accuracy.

## 4. Discussion

The 7- to 9-year-old children in this study demonstrated AV benefit to masked speech recognition thresholds. This finding is consistent with previous studies that have demonstrated AV benefit to word recognition accuracy in noise (Lalonde and Holt, 2016; Ross *et al.*, 2011) or with a competing talker (Knowland *et al.*, 2016) and sentence recognition accuracy in an SSN or a TTS (Lalonde and McCreery, 2020). However, results conflict with a previous study that used CRM stimuli with competing speech (Wightman *et al.*, 2006). In that study, 6- to 8.9-year-old children demonstrated no AV benefit. This discrepancy may be due to difference in level of task difficulty. Although the target stimuli of Wightman *et al.* (2006) were similar to the current study, the masker consisted of another CRM sentence spoken by a same-sex talker. Thus, children had to segregate two similar sentences and inhibit their response to the competing color-number combination, which may have affected children's ability to use cognitive resources to benefit from visual speech. In fact, the task was overall more difficult; children's mean 70.7% auditory threshold was approximately +5 dB SNR (compared to −1.8 dB for TTS in our study).

Although children demonstrated AV benefit in the TTS, the magnitude of benefit was no greater than in the SSN. In contrast, each adult who did not reach ceiling demonstrated greater AV benefit in the TTS than in the SSN, replicating previous findings (Avivi-Reich *et al.*, 2018; van Engen *et al.*, 2014; Helfer and Freyman, 2005). The results of the current study contrast with Lalonde and McCreery (2020), which showed that 6- to 12-year-old children and adults benefited five times more from visual speech on a syllable *detection* task in a TTS than in an SSN. This raises the question: why might children be able to use visual speech to segregate the target talker from the masker on a syllable detection task but not a sentence recognition task? The visual speech cues that improve syllable detection differ from those that improve sentence recognition. In AV syllable detection, visual speech temporally cues the onset or peak of the syllable (Bernstein *et al.*, 2004; Lalonde and Werner, 2019). These benefits are not specific to speech; visual pre-cues, such as a visual flash prior to the acoustic stimulus onset, afford similar benefits to tone detection in noise (low perceptual masking) and random-frequency two-tone maskers (high perceptual masking) in both children and adults (Bonino *et al.*, 2013).

In contrast to syllable detection, AV benefit to masked sentence recognition involves more than simple temporal cues. Listeners combine the cues extracted from the visual signal with the sparse acoustic information extracted from masked speech (Peelle and Sommers, 2015). Additionally, for connected speech in competing talker environments, ongoing cross-modality correlations between the envelope of target acoustic speech and preceding visible mouth movements help to perceptually amplify the target talker (Zion Golumbic *et al.*, 2013). Given the added complexity of AV cues that support sentence recognition, it is not surprising that children seem more adult-like in their use of visual speech as a grouping cue for syllable detection than sentence recognition.

A number of factors could explain why children failed to demonstrate added AV benefit in the TTS. One explanation is that children do not use visual speech to perceptually segregate sentences spoken by the target talker. Another potential explanation is that children use visual speech in this way, but (1) the acoustic-phonetic information that children obtain differs across the two maskers and (2) the improvement from phonetic information in the SSN happens to be approximately the same as the combined benefit from acoustic-phonetic information and perceptually segregating the talker in the TTS. Future studies might differentiate between these alternatives by assessing whether AV benefit decreases in the presence of other grouping cues (Helfer and Freyman, 2005) or by examining differences in phonetic supplementation across conditions and age groups using ideal time-frequency segregation (Kidd *et al.*, 2016).

Additional studies are needed to determine whether the current findings are generalizable. We effectively filtered out adults who were good speechreaders, as they could exceed the 70.7% threshold performance level for our stimulus set using only visual speech. Further the consistent temporal structure of the stimuli likely allowed participants to predict when target words would occur rather accurately with limited audibility. This potentially limited the benefit of temporal cues from ongoing cross-modal correlations. Such cues may be more informative for less predictable target sentences, increasing AV benefit in the TTS for listeners rely on these cues. Alternate test procedures—such as measuring full psychometric functions in each condition—are needed to better understand the potential influences of masker- and age-related differences in psychometric function slope (MacPherson and Akeroyd, 2014; Sobon *et al.*, 2019) on masked auditory-only and AV thresholds.

Individual differences in children's AV benefit in both maskers were similarly predicted by speechreading accuracy. This finding is consistent with a previous study which demonstrated that 4- to 12-year-old children's ability to speechread single words correlated with AV benefit to word recognition thresholds with a single competing talker (Knowland *et al.*, 2016). We expected to observe higher correlations between speechreading and AV benefit in the noise masker, but this was not the case. The similarity in the predictive power of visual speech across maskers lends support to the interpretation that children relied on similar cues for AV benefit in the SSN and in the TTS.

The results of this study contribute to the body of evidence indicating that children can use visual cues from talkers to better understand speech in noisy environments. The results demonstrate that adult–child differences in AV benefit to speech recognition are likely more pronounced in speech maskers than in steady-state noise maskers. Further, the results suggest that developmental differences in susceptibility to different forms of masking may interact in complex ways with the ability to use different AV speech cues. Additional studies are needed to determine the developmental time-course of the ability to benefit from visual speech in different types of maskers.

## Footnotes

^1^ Note the outlier in Fig. 1(C). This child benefited >30 dB in the SSN masker. This child performed near ceiling in the AV SSN condition, but not in the AV TTS condition. The same pattern of statistical results emerged when this child was excluded from analyses.
